# MicroRNA-216a-3p promotes sorafenib sensitivity in hepatocellular carcinoma by downregulating MAPK14 expression

**DOI:** 10.18632/aging.103670

**Published:** 2020-09-21

**Authors:** Zhong Wan, Tingyu Liu, Liang Wang, Rong Wang, Hai Zhang

**Affiliations:** 1 Department of Pharmacy, Shanghai First Maternity and Infant Hospital, Tongji University School of Medicine, Shanghai 201204, China; 2 Urologic Medical Center, Shanghai General Hospital, Shanghai Jiao Tong University School of Medicine, Shanghai 200080, China; 3 State Key Laboratory of Oncology in South China, Collaborative Innovation Center for Cancer Medicine, Sun Yat-Sen University Cancer Center, Guangzhou 510060, China; 4 Department of Pharmacy, Shanghai 9th People's Hospital, Shanghai Jiao Tong University School of Medicine, Shanghai 201999, China

**Keywords:** hepatocellular carcinoma, MAPK14, sorafenib resistance, miR-216a-3p

## Abstract

We investigated MAPK14-dependent resistance to sorafenib in hepatocellular carcinoma (HCC). Bioinformatics analysis and dual luciferase reporter assays in HCC cell lines showed that miR-216a-3p directly binds to the 3’UTR of MAPK14 mRNA and downregulates MAPK14 protein expression. Consequently, miR-216a-3p expression correlates inversely with MAPK14 protein levels in HCC patient tissues. miR-216a-3p overexpression significantly increases the sorafenib sensitivity of HCC cells by suppressing MAPK14 expression and reducing the subsequent activation of the MEK/ERK and ATF2 signaling pathways. The growth of xenograft tumors derived from miR-216a-3p-overexpression HCC cells was significantly diminished in sorafenib-treated Balb/c nude mice compared to controls. High miR-216a-3p levels in HCC tissue samples prior to treatment correlated with a better sorafenib response and favorable prognosis. Our findings thus demonstrate that miR-216a-3p enhances sorafenib sensitivity in HCC cells and tumor tissues by decreasing MAPK14 levels, thereby inhibiting the MAPK14-dependent MEK/ERK and ATF2 signaling.

## INTRODUCTION

Nearly 841,000 new cases and 782,000 deaths were reported because of liver cancer in 2018 according to the global cancer statistics [1]. The survival rates of liver cancer patients is low because it is highly invasions and metastasizes rapidly, and the symptoms are not obvious during early stages [2]. Therein, hepatocellular carcinoma (HCC) accounts for 90% of liver cancer cases and represents one of the most common malignant tumors of the digestive tract [3]. The overall prognosis of HCC patients is poor, and an understanding of this disease and its risk factors is crucial for screening at-risk individuals, early recognition, and timely diagnosis [4].

In the past few decades, there has been considerable progress in the early diagnosis and treatment of HCC. Currently, liver resection surgery and liver transplantation are the main treatments for HCC patients [5]. However, majority of HCC patients are diagnosed in advanced stages and are not amenable for surgical treatments. Moreover, the 5-year recurrence rate for patients with early and middle stage HCC that undergo radical surgery is very high [6, 7].

In treatment options, several chemotherapeutic drugs are available to treat HCC patients before or after surgery [8]. However, most chemotherapy drugs are not very effective because of the high rates of resistance of HCC cells against these drugs and high toxicity due to poor selectivity of the traditional chemotherapy drugs [9, 10]. Molecular targeted therapy has emerged as the treatment of choice for various malignancies including HCC and includes several tyrosine kinase inhibitors and monoclonal antibodies, which inhibit tumor cell growth by blocking specific tumor cell surface receptors, signaling pathways, and angiogenesis [11].

Sorafenib is a multi-targeted, small molecule tyrosine kinase inhibitor that blocks proliferation of tumor cells by inhibiting RAF/MEK/ERK and other signaling pathways and inhibits VEGF and PDGF receptors to suppress tumor-related angiogenesis [12]. Sorafenib is safe, well tolerated and highly effective in treating advanced HCC patients [13]. So far, the mechanisms of HCC patients develop primary or acquired resistance against sorafenib involve molecular level of tumor cells and the tumor stromal environment [14].

The activation of MAPK14 is involved in the multidrug resistance of hepatocellular carcinoma [15]. However, its upstream mechanism in sorafenib resistance of HCC cells is not clear. Therefore, we investigated the mechanisms that regulate MAPK14 protein expression and sorafenib resistance in HCC patients.

## RESULTS

### MiR-216a-3p enhances sorafenib resistance in HCC cells by decreasing the protein levels of MAPK14

**Figure 1:**
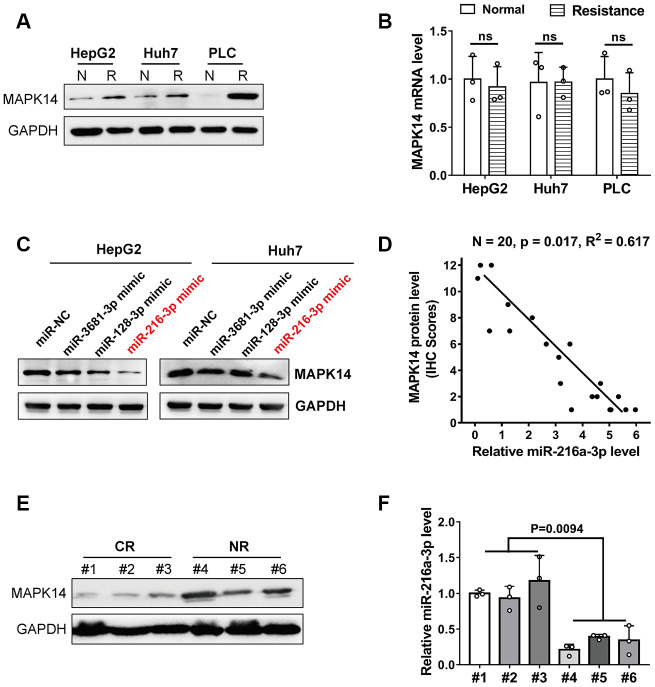
**MiR-216a-3p levels correlate with MAPK14 protein expression and sorafenib sensitivity in HCC cells and tumor tissues.** (**A**) Representative western blot images show MAPK14 protein expression in sorafenib-resistant and normal HCC cell lines. N: normal, R: resistance. GAPDH was used as loading control. (**B**) Q-PCR analysis shows relative MAPK14 mRNA levels in sorafenib-resistant and normal HCC cell lines. (**C**) Representative western blot shows MAPK14 protein expression in HCC cells transfected with miR-NC (negative control), miR3681-3p, miR128-3p and miR216a-3p mimics. GAPDH was used as loading control. (**D**) Pearson correlation analysis of MAPK14 protein and miR-216a-3p expression in 20 HCC patient tissue samples by IHC scores. (**E**) Representative western blot shows MAPK14 protein expression in tumor tissues from 3 CR (Complete response) to sorafenib and 3 NR (No response) to sorafenib HCC patients. (**F**) Q-PCR analysis shows relative miR-216a-3p levels in tumor tissues from 3 sorafenib-sensitive and 3 sorafenib-resistant HCC patients (n=3).

**Figure 2:**
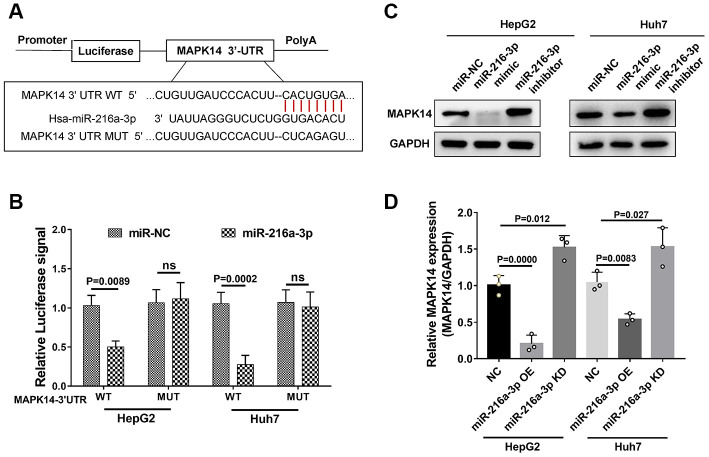
**MiR-216a-3p directly targets 3’UTR region of MAPK14 mRNA.** (**A**) Schematic representation shows potential miR-216a-3p binding sites in the WT and mutated 3’UTR of MAPK14 mRNA. (**B**) Dual luciferase reporter assay results show the luciferase activity from WT and mutant MAPK14-3’UTR luciferase constructs in HCC cells. (**C**) Representative western blot and (**D**) histogram plot shows relative MAPK14 protein expression in HCC cells transfected with miR-NC, miR-216a-3p mimic or miR-216a-3p inhibitor. GAPDH was used as loading control.

Western blot analysis showed that MAPK14 expression was significantly up-regulated in sorafenib-resistant HCC cell lines compared to normal HCC cells lines (Huh-7, HepG2, and PLC/PRF/5 cells, Figure 1A and Supplementary Figure 1). However, MAPK14 mRNA levels were similar in both sorafenib-resistant and normal HCC cells (Figure 1B). These results suggest a post-transcriptional regulation of sorafenib resistance in HCC cells. Since microRNAs (miRNAs) modulate protein levels post-transcriptionally, we searched the Targetscan databases to identify miRNAs that bind to 3’UTR of MAPK14 mRNA. We identified miR-3681-3p, miR-128-3p and miR-216-3p as potential miRNAs targeting MAPK14 mRNA (Supplementary Table 1). Among these, western blot analysis showed that MAPK14 protein levels were significantly downregulated in HCC cell lines transfected miR-216-3p mimic (Figure 1C). To identify the miR-216a-3p levels responsible for sorafenib unresponsiveness, we performed a clinical analysis in a set of pre-treated tumor tissues from 20 patients randomly, the IHC scores showed miR-216-3p expression negatively correlated with MAPK14 protein levels in clinical HCC and adjacent normal liver tissues (n=20, p=0.017; Figure 1D). Meanwhile, we measured the levels of MAPK14 protein on pre-treated tumor tissues from a cohort of HCC patients which presented with good or poor responses to sorafenib treatment, the result showed MAPK14 level were significantly higher in sorafenib-resistant (No Response) HCC patients compared to sorafenib-responsive (Complete Response) patients (Figure 1E and Supplementary Table 2). Moreover, miR-216a-3p levels were significantly reduced in sorafenib-resistant HCC patients compared to sorafenib-sensitive HCC patients (Figure 1F). These results suggest that miR-216a-3p regulates MAPK14 protein levels in HCC tissues post-transcriptionally. Dual luciferase reporter assay confirmed that miR-216a-3p directly binds to the wild-type 3'-UTR sequence of MAPK14 mRNA and does not bind mutated 3’UTR sequence (Figure 2A, 2B). Furthermore, western blot analysis showed that MAPK14 protein levels were significantly reduced in miR-216a-3p overexpression HCC cells and significantly increased in miR-216a-3p knockdown (KD) HCC cells compared to their corresponding controls (Figure 2C, 2D). These results confirmed that miR-216a-3p inhibits MAPK14 protein levels in HCC cells by binding to the 3’UTR of MAPK14 mRNA.

### MiR-216a-3p enhances sorafenib sensitivity of HCC cells

**Figure 3:**
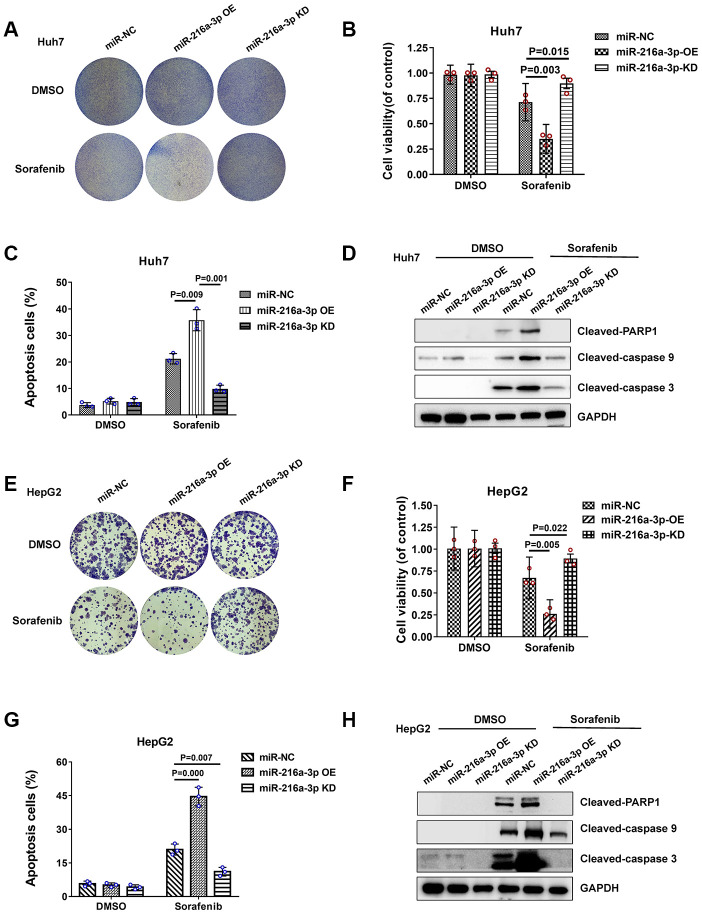
**Sorafenib treatment response of miR-216a-3p-overexpression and knock down HCC cells.** (**A**) Representative colony formation images show crystal violet staining of DMSO or sorafenib-treated NC-, miR-216-3a OE-, and miR-216a-3p KD-Huh-7 cells. (**B**) MTT assay results show viability of DMSO or sorafenib-treated NC-, miR-216-3a OE-, and miR-216a-3p KD-Huh-7 cells. (**C**) Flow cytometry assay results show percentage apoptosis in DMSO or sorafenib-treated NC-, miR-216-3a OE-, and miR-216a-3p KD-Huh-7 cells. (**D**) Representative western blot images show cleaved-PARP1/caspase9/caspase3 levels in DMSO or sorafenib-treated NC-, miR-216-3a OE-, and miR-216a-3p KD-Huh-7 cells. (**E**) Representation colony formation images show crystal violet staining of DMSO or sorafenib-treated NC-, miR-216-3a OE-, and miR-216a-3p KD-HepG2 cells. (**F**) MTT assay results show viability of DMSO or sorafenib-treated NC-, miR-216-3a OE-, and miR-216a-3p KD-HepG2 cells. (**G**) Flow cytometry assay results show percentage apoptosis in DMSO or sorafenib-treated NC-, miR-216-3a OE-, and miR-216a-3p KD-HepG2 cells. (**H**) Representative western blot images show cleaved-PARP1/caspase9/caspase3 levels in DMSO or sorafenib-treated NC-, miR-216-3a OE-, and miR-216a-3p KD- HepG2 cells.

Next, we analyzed if miR-216a-3p regulates sorafenib sensitivity of HCC cells. Colony formation assay results showed that sorafenib-treated miR-216a-3p overexpression (OE) significantly reduced colony formation and sorafenib-treated miR-216a-3p KD significantly increased colony formation in Huh-7 and HepG2 cell lines compared to corresponding controls (Figure 3A and 3E). MTT cell viability assay showed that sorafenib-treated miR-216a-3p OE significantly reduced cell viability, whereas, sorafenib-treated miR-216a-3p KD significantly increased cell viability in Huh-7 and HepG2 cells compared to corresponding controls (Figure 3B and 3F). Flow cytometry analysis showed that sorafenib-treated miR-216a-3p OE significantly increased apoptosis, whereas, sorafenib-treated miR-216a-3p KD significantly decreased apoptosis in Huh-7 and HepG2 cells compared to the corresponding controls (Figure 3C, Supplementary Figure 2A and Figure 3G, Supplementary Figure 2B). In addition, western blot analysis confirmed significant increase in PARP-1, caspase-9 and caspase-3 cleavage protein in sorafenib-treated miR-216a-3p OE groups, whereas, sorafenib-treated miR-216a-3p KD significantly decreased in Huh-7 and HepG2 cells compared to the corresponding controls (Figure 3D and 3H). However, apoptosis ratio and degree of miR-216a-3p OE and KD HCC cells in the absence of sorafenib treatment was similar (Figure 3C, 3G). Taken together, these results show that high miR-216a-3p levels increase sorafenib sensitivity in HCC cells.

### MiR-216a-3p enhances sorafenib sensitivity by decreasing the protein levels of MAPK14 in HCC cells

**Figure 4:**
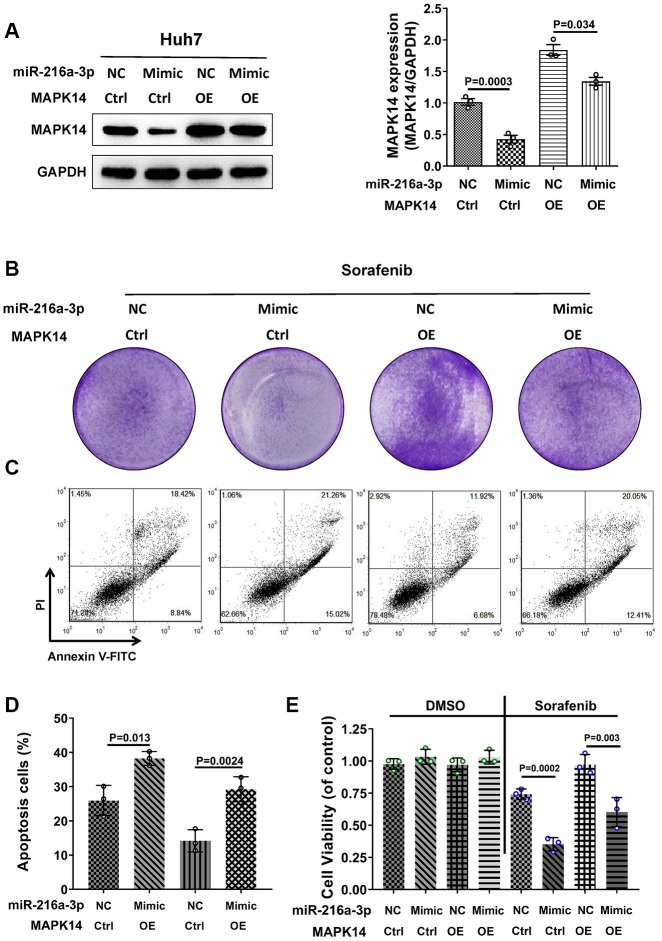
**MiR-216a-3p regulates sorafenib sensitivity in HCC cells by decreasing the protein levels of MAPK14.** (**A**) Representative western blot shows MAPK14 protein expression in control and sorafenib-treated miR-216-3p OE, MAPK14 OE or miR-216a-3p OE plus MAPK14 OE Huh-7 cells. (**B**) Colony formation assay results of control and sorafenib-treated miR-216-3p OE, MAPK14 OE or miR-216a-3p OE plus MAPK14 OE Huh-7 cells. (**C**, **D**) Flow cytometry assay shows percentage apoptosis in control and sorafenib-treated miR-216-3p OE, MAPK14 OE or miR-216a-3p OE plus MAPK14 OE Huh-7 cells. (**E**) MTT assay results show viability of control and sorafenib-treated miR-216-3p OE, MAPK14 OE or miR-216a-3p OE plus MAPK14 OE Huh-7 cells.

To further demonstrate that miR-216a-3p regulates sorafenib resistance of HCC cells through MAPK14, we performed a functional rescue experiment by overexpressing miR-216a-3p and MAPK14 alone or in combination in Huh-7 cells. Western blot analysis showed that sorafenib-treated miR-216a-3p OE significantly down-regulated MAPK14 protein levels, whereas, sorafenib-treated MAPK14 OE significantly increased MAPK14 protein levels (Figure 4A). However, MAPK14 protein overexpression was reduced by miR-216a-3p in miR-216a-3p OE plus MAPK14 OE Huh-7 cells (Figure 4A). Subsequently, colony formation assay showed that sorafenib sensitivity was highest for miR-216a-3p OE Huh-7 cells followed by miR-216a-3p OE plus MAPK14 OE Huh-7 cells, whereas, MAPK14 OE Huh-7 cells were resistant to sorafenib (Figure 4B). Flow cytometry assay results showed that the apoptotic rates of miR-216a-3p OE plus MAPK14 OE Huh-7 cells were significantly higher than MAPK14 OE Huh-7 cells, but, lower than miR-216a-3p OE Huh-7 cells (Figure 4C–4E). However, MTT assay results confirmed that overexpression of miR-216a-3p and MAPK14 alone or in combination did not alter the proliferation of Huh-7 cells in the absence of sorafenib treatment (Figure 4D). These data demonstrate that miR-216a-3p increases sorafenib sensitivity of HCC cells by decreasing MAPK14 protein expression.

### MiR-216a-3p enhances sorafenib sensitivity by attenuating MAPK14-dependent MEK/ERK and ATF2 signaling pathways in HCC cells

**Figure 5:**
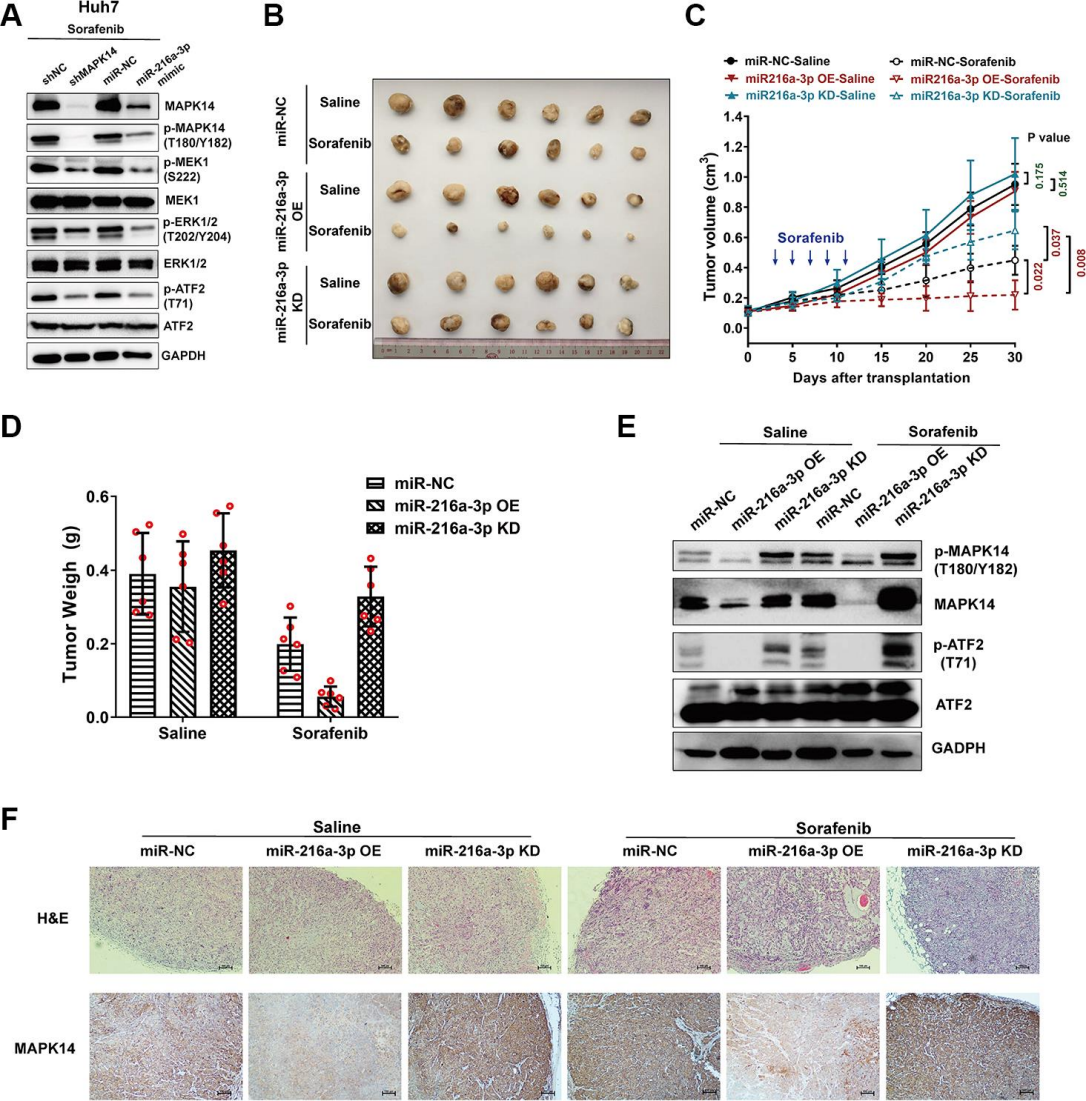
**MiR-216a-3p enhances sorafenib sensitivity in the xenograft HCC tumor mouse model by attenuating MAPK14-dependent MEK-ERK and ATF2 signaling pathways.** (**A**) Representative western blots show phospho-MEK1, MEK1, phospho-Erk1/2 and Erk1/2, phospho-ATF2 and ATF2 levels in sorafenib-treated Huh-7 cells transfected with shRNA-NC (negative control), shRNA-MAPK14, miR-NC (negative control), miR-216a-3p mimic respectively. (**B**) Comparison of saline or sorafenib treatment efficacy using Balb/c nude mice with xenograft tumors after injecting miR-NC, miR-216a-3p OE or miR-216a-3p KD Huh-7 cells. (**C**) The tumor size measurements and (**D**) tumor weight in saline or sorafenib-treated miR-NC, miR-216a-3p OE or miR-216a-3p KD groups of mice. (**E**) Western blot analysis show phospho-MAPK14, MAPK14, phospho-ATF2 and ATF2 levels in xenograft tumor tissues from saline or sorafenib-treated miR-NC, miR-216a-3p OE or miR-216a-3p KD groups of mice. (**F**) Representative IHC images show MAPK14 protein expression in xenograft tumor tissue sections from saline or sorafenib-treated miR-NC, miR-216a-3p OE or miR-216a-3p KD groups of mice. Also shown are H&E stained xenograft tumor tissue sections from saline or sorafenib-treated miR-NC, miR-216a-3p OE or miR-216a-3p KD groups of mice.

Next, we analyzed the MAPK signaling pathway to determine the mechanism through which miR-216a-3p sensitizes HCC cells to sorafenib treatment via MAPK14. Western blot analysis showed that phospho-MEK1, phospho-Erk1/2 and phospho-ATF2 levels were significantly reduced in sorafenib-treated miR-216a-3p OE and MAPK14 KD Huh-7 cells and significantly increased in the sorafenib-treated miR-216a-3p KD Huh-7 cells, compared to the corresponding controls (Figure 5A). This demonstrates that miR-216a-3p increases sorafenib sensitivity of Huh-7 cells by suppressing MAPK14-dependent MEK/ERK and ATF2 signaling pathways.

### MiR-216a-3p promotes tumor response to sorafenib treatment in the nude mice xenograft model

Next, we analyzed if miR-216a-3p enhances sorafenib-sensitivity of HCC tumors in the nude mice xenograft model. We subcutaneously injected miR-216a-3p OE or KD Huh-7 cells into BALB/c nude mice and analyzed xenograft tumor growth after transplantation for 30 days. Sorafenib treatment was performed by oral gavage every 2 day (×5 times) from 3^th^ day after transplantation. The growth of tumors in saline-treated BALB/c nude mice xenografted with control, miR-216a-3p OE, and miR-216a-3p KD Huh-7 cells were similar (Figure 5B, 5C). This showed that miR-216a-3p OE or KD did not affect tumor growth in the absence of sorafenib. On the other hand, tumors derived miR-216a-3p KD Huh-7 cells were significantly larger compared to those derived from miR-216a-3p OE Huh-7 cells as well as control Huh-7 cells in sorafenib-treated BALB/c nude mice (Figure 5B, 5C). Furthermore, the tumor inhibition ratio (sorafenib treatment effect) was significantly enhanced in the miR-216a-3p OE groups compared to the miR-negative control (NC) groups (84.17% versus 48.97%), conversely, the sorafenib treatment effect was markedly attenuated in the miR-216a-3p KD groups compared to the miR-NC groups (27.56% versus 48.97%) (Figure 5D). In addition, western blot analyses showed that phospho-MAPK14, MAPK14, phospho-ATF2 and levels were significantly reduced in miR-216a-3p OE xenograft tumors and significantly increased in the miR-216a-3p KD xenograft tumors compared to the control group with or without sorafenib treatment (Figure 5E). IHC and H&E staining assay results showed that in comparison with the control group, sorafenib-treated miR-216a-3p OE group tumors showed reduced MAPK14 staining and increased inflammatory cell infiltration, whereas, sorafenib-treated miR-216a-3p KD tumors showed increased MAPK14 staining and decreased inflammatory cell infiltration (Figure 5F). These results demonstrate that miR-216a-3p promotes sorafenib sensitivity of HCC tumors by suppressing MAPK14-dependent activation and ATF signaling pathways *in vivo*.

### High miR-216a-3p levels are associated with tumor response and favorable prognosis in HCC patients treated with sorafenib

**Figure 6:**
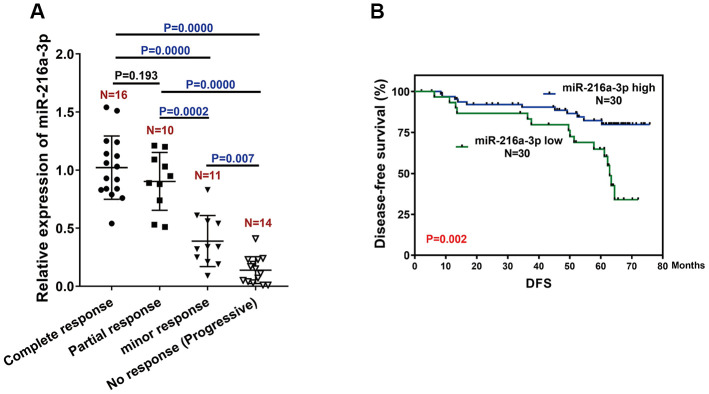
**MiR-216-3p expression correlates with treatment response and prognosis of HCC patients treated with sorafenib.** (**A**) Q-PCR analysis shows miR-216a-3p levels in pretreatment tumor tissues (biopsy) from patients showing different (poor, moderate or high) response to sorafenib treatment. As shown, patients with good response have significantly higher miR-216a-3p expression compared to those with poor response. (**B**) Kaplan Meier survival curve analysis shows disease-free survival of HCC patients with high or low miR-216-3p expression in the tumor tissues. As shown, DFS rates are significantly higher for HCC patients with high miR-216a-3p levels compared to those with low miR-216a-3p levels.

Next, we analyzed the relationship between miR-216a-3p levels and tumor regression after sorafenib treatment by estimating miR-216a-3p expression levels in pretreatment biopsy tumor specimens from 51 HCC patients who received sorafenib with or without surgery. The miR-216a-3p levels were significantly higher in HCC patients that showed a positive response to sorafenib treatment compared to those with minimal or no response to sorafenib treatment (Figure 6A).

We performed ROC curve analysis and classified patients into high or low miR-216a-3p expression groups using a cut-off value of 1.9. Kaplan–Meier survival curve analysis showed that disease-free survival (DFS) was significantly longer in sorafenib-treated patients with high miR-216a-3p levels compared to those with low miR-216a-3p expression (Figure 6B, p=0.002). These results demonstrate that high miR-216a-3p levels indicate favorable tumor regression and prognosis in sorafenib-treated HCC patients.

## DISCUSSION

Hepatocellular carcinoma is the sixth most prevalent cancer and the third most frequent cause of cancer-related death [16]. Although HCC treatments have greatly improved in the recent decades, the prognosis remains poor for several HCC patients because of late diagnosis and high recurrence rates [17, 18]. Recent studies have shown that microRNAs regulate drug resistance mechanisms and tumor progression by regulating the expression of proteins that modulate tumor growth and progression [19, 20].

In HCC patients, constitutive activation of the RAS/ERK signaling pathway promotes tumor growth, progression and recurrence [21]. Sorafenib, a RAS/ERK pathway inhibitor, is currently the most effective treatment of early and advanced HCC patients [22–24]. Two randomized placebo-controlled phase III clinical trials showed that sorafenib treatment delayed disease progression in advanced HCC patients by 2.8 months and extended overall survival by 2.3 months [25, 26]. However, drug resistance is commonly encountered against sorafenib treatment in HCC patients. The mechanisms of sorafenib-related drug resistance are not clear. Hence molecular markers that can predict treatment response to sorafenib are not available [27]. As a result, several HCC patients are treated with sub-optimal sorafenib doses to overcome treatment-related adverse events and in serious cases, treatment is completely stopped [28, 29]. Therefore, understanding the mechanisms regulating sorafenib-related resistance is necessary to improve the prognosis of HCC patients.

In several cancers, multidrug mechanisms involve activation of MAPK14 [30, 31]. The aberrant expression of MAPK14 triggers pro-apoptotic and pro-inflammatory mechanisms in several human diseases including cancers [32, 33]. Our study demonstrates that MAPK14 protein expression is significantly upregulated in sorafenib-resistant HCC tumor samples, but, MAPK14 mRNA levels are normal. This indicates post-transcriptional regulation of MAPK14. The microRNAs are a class of non-coding RNAs that are approximately 22 nucleotides long, and inhibit protein translation by base pairing with the 3’-UTR sequences of the target mRNAs, thereby regulating cellular differentiation, survival and tumorigenesis [34, 35]. In this study, we identified miR-216a-3p as a potential post-transcriptional regulator of MAPK14 through bioinformatics analysis. Furthermore, dual luciferase reporter assay confirmed that miR-216a-3p binds to the 3’UTR of MAPK14. We also demonstrate that miR-216a-3p expression is significantly down-regulated in sorafenib-resistant HCC tumor tissue samples.

Recent studies in HCC patients have shown that molecular targeted therapy of sorafenib-resistant pathways in combination with sorafenib is more effective than sorafenib treatment alone [36, 37]. We demonstrate that high miR-216a-3p levels promote sorafenib sensitivity by suppressing MAPK14 protein levels in miR-216a-3p OE and MAPK14 KD Huh-7 cells. Furthermore, miR-216a-3p OE in HCC cells promotes sorafenib sensitivity in the xenograft tumor nude mice model by downregulating MAPK14 protein levels and inhibiting activation of MEK/ERK and ATF signaling pathways.

In conclusion, our study demonstrates that miR-216a-3p levels correlate with sorafenib sensitivity in HCC tumor tissues. MiR-216a-3p downregulates MAPK14 protein levels by binding to its 3’-UTR and subsequently inhibits the activation of MAPK14-dependent MEK/ERK and ATF signaling pathways. Hence, our study demonstrates that miR-216a-3p is a potential prognostic indicator and therapeutic target for HCC patients.

## MATERIALS AND METHODS

### Cell culture

Huh-7, HepG2, and PLC/PRF/5 cells were obtained from the Shanghai Institute of Biochemistry and Cell Biology (Shanghai, China). Huh-7 and PLC/PRF/5 cells were cultured in DMEM medium, whereas, HepG2 cells were grown in MEM medium. Both media were supplemented with 10% FBS, 1 mM sodium pyruvate, and 1% Penicillin and streptomycin. The cells were cultured in a humidified incubator at 37°C and 5% CO_2_. Sorafenib resistant Huh-7, HepG2 and PLC/PRF/5 cells were generated by treating parental cells with stepwise increasing (1, 2.5, 5, 20 μM) doses of sorafenib (Y0002098, St. Louis, MO, USA). All cell lines were authenticated by short tandem repeat analysis at the China Center for Type Culture Collection. The cell lines were kept frozen in liquid nitrogen and used for experiments between passages 3 and 10 after thawing.

### Cell transfection

Transfection experiments with miRNA mimics, miRNA inhibitors, siRNAs (RiboBio, Guangzhou, China), and their corresponding controls were carried out with 60-70% confluent cells grown in 6-well plates using Lipofectamine 2000 (Invitrogen, Carlsbad, CA, USA) according to manufacturer's instructions. Transient plasmid transfections were performed using Lipofectamine 3000 (Invitrogen, Carlsbad, CA, USA) according to the manufacturer's protocol. The control and MAPK14-specific shRNAs (Supplementary Table 3) were cloned into the pLKO.1 vector (Sigma-Aldrich, St. Louis, MO, USA) according to the manufacturer’s instructions (plasmid sequencing data not shown). The plasmids were co-transfected into 293T cells with the lentiviral packaging vector (FulenGen, Guangzhou, China) to obtain recombinant lentiviruses. Control and stable MAPK14 knockdown Huh-7 cells were selected using puromycin.

To further validate the direct targeting of MAPK14 by miR-216a-3p, we performed a functional rescue experiment by co-transfecting Huh-7 cells with the miR-216a-3p mimic and plasmid constructs expressing MAPK14 (pcDNA-MAPK14; Genechem, USA) using Lipofectamine 2000 (Invitrogen, USA). DNA sequencing analysis was used to confirm the complete MAPK14 coding regions in the plasmid construct.

### HCC patient specimens

Freshly frozen and paraffin-embedded HCC tissues were obtained from the Sun Yat-Sen University Cancer Center (SYSUCC, Guangzhou, China). Our study included diagnosed 60 HCC patients who received sorafenib treatment between May 2008 and May 2016 at SYSUCC (Supplementary Table 4). Among the 60 HCC tissues, 20 HCC tissues were randomly obtained to analysis miR-216a-3p and MAPK14 expression levels. Also among the 60 HCC tissues, 51 HCC patients’ overall tumor response to sorafenib were analyzed, which was scored as a complete response (CR), partial response (PR), minor response (MR; reduction in tumor size of ≥ 25% but<50%) or No response (Progressive). The study was approved by the SYSUCC Ethics Committee (Approval number: GZR2016-172) and conducted in accordance with the Declaration of Helsinki. We obtained written informed consent from all patients.

### Nude mice xenograft tumor model

All animal experiments were approved by the Administrative Committee of Experimental Animal Care and Use of Tongji University School of Medicine and conformed to the National Institute of Health guidelines on the ethical use of animals (TJLAC-019-126).

Stable transfection with constructed lentivirus Plasmid, LV3-miR-negative control (NC), LV3-miR-216a-3p mimic (OE) and LV3-miR-216a-3p inhibitors (KD) in Huh7 cells. Twenty adult male BALB/c nude mice weighing 20 ± 2 g were obtained from the Model Animal Research Center of Nanjing University (Nanjing, China). We divided the mice randomly into six groups: (1) miR-NC (Negative control) + Saline; (2) miR-216a-3p OE + Saline; (3) miR-216a-3p KD + Saline; (4) miR-NC + Sorafenib; (5) miR-216a-3p OE + Sorafenib; and (6) miR-216a-3p KD + Sorafenib. We injected mice subcutaneously with 1×10^6^ Huh-7 cells in 100 μL PBS to generate xenograft tumors in mice. The size of tumors was measured every 5 days and the tumor volume (mm^3^) was calculated as (width) ^2^ × (length) /2. For drug administration, mice were treated with 100 mg/kg body weight sorafenib. Sorafenib was dissolved in a 4×cremophor EL/95% ethanol solution (50:50). Treatment was performed by oral gavage every 2 day (20mg/kg × 5) from 3^th^ day after transplantation. The mice were sacrificed on the 30^th^ day after transplantation and tumor tissues were harvested weighed and subjected to analysis.

### Real-time quantitative PCR

Total RNA from the tissue samples or cell lines was extracted using TRIzol reagent (Invitrogen). The quality and quantity of the RNA samples was assessed using the Agilent 2100 Bioanalyzer and NanoDrop ND-1000 Spectrophotometer (Agilent, Santa Clara, CA, USA). Complementary DNA (cDNA) synthesis was performed using the M-MLV Reverse Transcriptase kit (Promega, Madison, WI, USA). Then, equal amount of cDNA samples were subjected to quantitative PCR (qPCR) using iTaq SYBR Green Mix (Bio-Rad, Hercules, CA, USA) and specific primers (Supplementary Table 5). The relative levels of the specific mRNAs were determined by calculating 2^−ΔCt^ using GAPDH mRNA levels as the internal control.

For miRNA detection, total RNA samples were extracted form cells or tissues using miRNeasy Mini Kit (Qiagen, Dusseldorf, Germany). Then, reverse transcription and quantitative RT-PCR was carried out in a two-step reaction using the NCode™ VILO™ miRNA cDNA Synthesis and EXPRESS SYBR® GreenER™ miRNA Q-PCR (Invitrogen, CA) kits, respectively, according to the manufacturer’s instructions. The sequence-specific forward primers for the mature hsa-miR-216a-3p and U6 internal control are listed in Supplementary Table 5.

The miR-216a-3p levels in HCC patient samples were independently and semiquantitatively assessed by two pathologists. The relative miR-216a-3p expression was converted into an immunoreactive score (IRS) score, which ranged from 0 to 4. Receiver operating characteristic (ROC) curve was used to determine the cut-off value for miR-216a-3p levels, and the patients were categorized into low and high miR-216a-3p expressing groups.

### MTT cell proliferation assay

The MTT assay was used to determine the status of HCC proliferation in different experimental groups. The cells were grown for 48 h and then incubated for another 4 h at 37 °C with 200 μl of MTT solution. The optical density was read at 490 nm using a microplate reader.

### Colony formation assay

The control or transfected Huh-7 or HepG2 cells were seeded in six-well plates at the density of 600 cells per well and grown for 2 weeks in DMEM medium containing 10% FBS. Then, the medium was removed, and the cells were stained with crystal violet for 35 min. The colonies were counted under a light microscope.

### Flow cytometry

Huh7 or HepG2 cells (1×10^5^/well) were harvested after growing for 48 h in 5% FBS-supplemented DMEM medium. Then, the cells were washed in pre-cold PBS, fixed in 70% cold ethanol at 4 °C for 4 h, centrifuged at 1000 rpm for 10 min and re-suspended in 1× pre-chilled PBS. Then, the cells were stained with Annexin V-FITC and PI according to the manufacturer’s instructions and analyzed in a BD FACS cytometer. The percentages of apoptotic cells (Annexin-V ^+^ PI^+^ and Annexin-V ^+^ PI^-^) were determined in all samples.

### Western blot

Total protein samples were prepared by lysing cells and tissues in RIPA buffer and quantified using the BCA method. Equal amounts of total protein were subjected to electrophoresis at 120 V for 1.5 h in a 10% Bis-Tris gel. Then, the proteins were transferred onto PVDF membranes at 320 mA for 1 h. The PVDF membranes were blocked by incubating with 5% BSA at room temperature for 1.5 h. Then, the blots were incubated overnight with primary antibodies at 4°C, followed incubation with the HRP-conjugated secondary antibody (1:5000 dilution) for 1 h. The membranes were developed using enhanced chemiluminescence (ECL) method and the relative levels of various proteins were detected using GAPDH as loading control. The primary antibodies used in this study are: p38α or MAPK14 (Cell Signaling; Cat. No. 9218; 1:1,000), GAPDH (Beyotime Biotechnology, Nantong, China; Cat. No. AG019; 1:1000), phospho-MAPK14 (Thr180/Tyr182; Cell Signaling; Cat. No. 4511 (D3F9); 1:1,000), ERK1/2 (Cell Signaling; Cat. No. 4695 (137F5); 1:1,000), phospho-p44/42 ERK1/2 (Thr202/Tyr204; Cell Signaling; Cat. No. 4370 (D13.14.4E); 1:1,000); MEK1 (Cell Signaling; Cat. No. 2352 (61B12); 1:1,000), phospho-MEK1 (Ser222; Santa Cruz Biotechnology; Cat. No. sc-293106; 1:200); ATF2 (Cell Signaling; Cat. No. 35031 (D4L2X); 1:1,000), phospho-ATF2 (Thr71; Cell Signaling; Cat. No. 9221; 1:1,000).

### Dual luciferase reporter assay

We seeded 1×10^5^ Huh-7 and HepG2 cells in 6-well plates for 24 h and transfected them with the wild-type or mutant MAPK14-3′UTR containing pGL3 luciferase plasmid (WT/Mut) or the control pGL3 luciferase plasmid and pRL-TK Renilla plasmid (1 ng) using Lipofectamine 3000 (Invitrogen, USA) according to the manufacturer’s recommendations (plasmid sequencing data not shown). Dual luciferase reporter system was used to analyze the luciferase activities after 48 h.

### Immunohistochemistry

The paraffin-embedded tumor tissue samples were cut into 5-μm thick sections, placed on polylysine coated slides, and deparaffinized in xylene. The specimens were rehydrated using a series of graded ethanol. Heat-induced antigen retrieval was performed in sodium citrate buffer (pH 6.0). Then, the samples were blocked with 10% goat serum before incubating overnight with primary anti-p38α/MAPK14 (1:200; Cat. No. 9218; Cell Signaling, USA) or the IgG isotype control in a humidified container at 4°C. Then, immunohistochemical staining was performed using the Dako Envision Plus System (Dako, Carpinteria, CA) according to manufacturer’s instructions.

### Statistical analyses

The data is reported as means ± SEM from three or more independent experiments. The differences between groups were compared using Student's t-test or one-way ANOVA. All statistical analyses were performed using the SPSS 16.0 software (SPSS Inc., USA). The χ^2^ and Fisher’s exact tests were used to analysis. The significance of the variables was tested using multivariate Cox regression and logistic regression models. Disease-free survival was defined as the interval between surgical resection and recurrence, metastasis, or the end of follow-up. Values of P < 0.05 were considered significant. Pearson's correlation analysis was used to evaluate the association between MAPK14 and miR-216a-3p levels.

## Supplementary Material

Supplementary Figures

Supplementary Table 1

Supplementary Tables 2 - 5
