# Supplementary material for: MicroRNA-216a-3p promotes sorafenib sensitivity in hepatocellular carcinoma by downregulating MAPK14 expression
Source: Aging (Albany NY). 2020 Sep 21;12(18):18192–208. doi: 10.18632/aging.103670 (PMC7585128; doi:10.18632/aging.103670)
Supplement: Supplementary Figures [file aging-12-103670-s003..pdf]

## SUPPLEMENTARY FIGURES

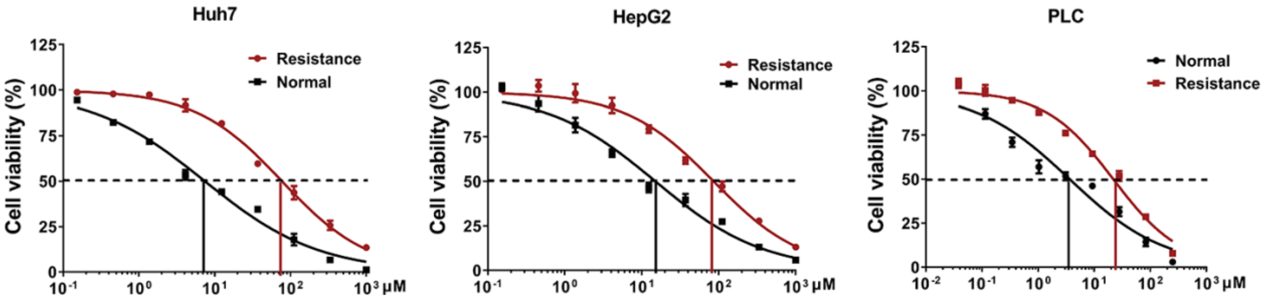


**Supplementary Figure 1. The effects of Sorafenib on the resistant or normal HCC cells.** MTT assays for cell proliferation inhibition test. Representative dose-response curves of sorafenib on Huh7/HepG2/PLC cells.

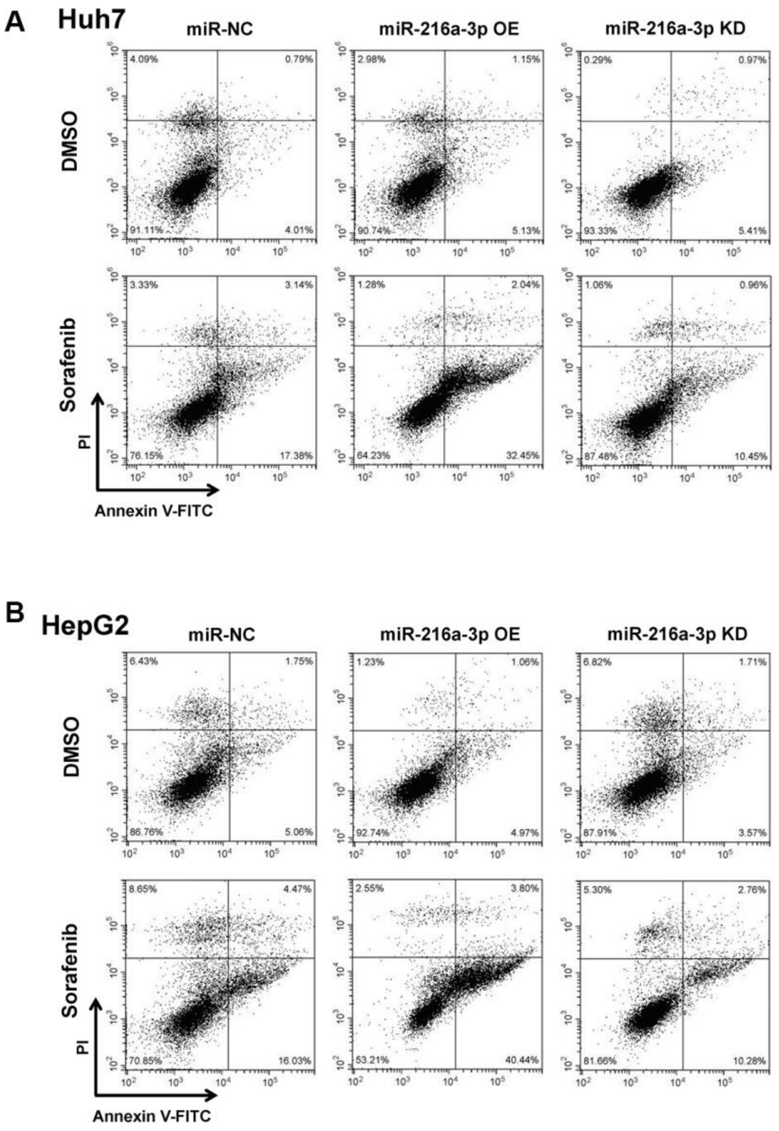


**Supplementary Figure 2.** Annexin V/PI double staining assays for apoptosis (representative charts of flow cytometric analysis) in DMSO or sorafenib-treated NC-, miR-216-3a OE-, and miR-216a-3p KD (**A**) Huh7 and (**B**) HepG2 cells with sorafenib treatment for 48h.
