# Supplementary material for: MicroRNA-216a-3p promotes sorafenib sensitivity in hepatocellular carcinoma by downregulating MAPK14 expression
Source: Aging (Albany NY). 2020 Sep 21;12(18):18192–208. doi: 10.18632/aging.103670 (PMC7585128; doi:10.18632/aging.103670)
Supplement: Supplementary Tables 2 - 5 [file aging-12-103670-s002..pdf]

SUPPLEMENTARY TABLES

Supplementary Table 2. Clinical characteristics of HCC patients for MAPK14 and miR-216a-3p level.

Patient ID	Age	Gender	TNM classification	Treatment	Months of treatment	Response
1	46	M	IIIA/ IIIB	sorafenib	6	CR
2	50	F	IIIA/ IIIB	sorafenib	8	CR
3	67	M	IIIA/ IIIB	sorafenib	7	CR
4	39	M	IIIA/ IIIB	sorafenib	8	NR
5	54	F	IIIA/ IIIB	sorafenib	5	NR
6	75	M	IIIA/ IIIB	sorafenib	11	NR

Abbreviation: CR, complete response; NR, no response (progression).

Supplementary Table 3. The information of shRNAs used in this study.

Target gene	Name	Position	Target sequence
Scramble	shNC	/	3'-GCGACGAUCUGCCUAAGAU-3'
MAPK14	shMAPK14	CDS	5'-GCTGAATTGGATGCACTATAA-3'

Supplementary Table 4. Correlation of clinicopathological parameters and miR-216a-3p expression (n=60).

Variables	No. of patients (%)	MiR-216a-3p expression		χ^2 test *p* value
		Low (n=30)	High (n=30)	
Gender				0.405
Female	37 (61.7)	19 (31.7)	18 (30.0)	
Male	23 (38.3)	11 (18.3)	12 (20.0)	
Age (mean:49y)				0.134
≤49y	27 (45.0)	13 (21.7)	14 (23.3)	
>49y	33 (55.0)	17 (28.3)	16 (26.7)	
Positive hepatitis status				0.595
Hepatitis B	39 (65.0)	15 (25.0)	24 (40.0)	
Hepatitis C	17 (28.3)	11 (18.3)	6 (10.0)	
TNM classification				**0.048***
II	3 (5.0)	1 (1.6)	2 (3.4)	
IIIA/ IIIB	36 (60.0)	22 (36.7)	14 (23.3)	
IV	21 (35.0)	7 (11.7)	14 (23.3)	
Child-Pugh status				0.573
A	43 (71.7)	12 (20.0)	31 (51.7)	
B	17 (28.3)	9 (15.0)	8 (13.3)	
Status				**0.014***
Alive	33 (55.0)	23 (38.3)	10 (16.7)	
Death	27 (45.0)	7 (11.6)	20 (33.4)	

*For analysis of correlation between miR-216a-3p levels and clinical features. Results were considered statistically significant at p<0.05.

Supplementary Table 5. Primers for qRT-PCR analysis.

Gene	Forward primer (5'-3')	Reverse primer (5'-3')
Hsa-miR-216a-3p	TCACAGTGGTCTCTGGGATTAT	Universal qPCR Primer (Invitrogen)
U6	CTCGCTTCGGCAGCACA	Universal qPCR Primer (Invitrogen)
MAPK14	CGAGCGTTACCAGAACCTGT	TCAGATCTGCCCCCATGAGA
GAPDH	GAAGGTGAAGGTCGGAGTC	GAAGATGGTGATGGGATTTC
